# A polycentric, randomized, parallel-group, study on Lertal®, a multicomponent nutraceutical, as preventive treatment in children with allergic rhinoconjunctivitis: phase II

**DOI:** 10.1186/s13052-019-0678-y

**Published:** 2019-07-18

**Authors:** Gianluigi Marseglia, Amelia Licari, Salvatore Leonardi, Maria Papale, Anna Maria Zicari, Laura Schiavi, Giorgio Ciprandi, Italian Study Group on Pediatric Allergic Rhinoconjunctivitis, F. Cardinale, S. Cherubini, P. Giordano, P. Marchisio, A. Martelli, D. Minasi, M. Miraglia del Giudice, F. Paravati, G. Pellegrini, A. Podestà, L. Pogliani, C. Salpietro, M. A. Tosca, A. Verrotti

**Affiliations:** 1 Pediatrics Clinic, Pediatrics Department, Policlinico San Matteo, University of Pavia, Pavia, Italy; 2 Department of Clinical and Experimental Medicine, University of Catania, Catania, Italy; 3 Pediatrics Department , Umberto I Hospital, Roma, Sapienza University, Rome, Italy; 4 Allergy Clinic, Casa di Cura Villa Montallegro, Via P. Boselli 5, 16146 Genoa, Italy

**Keywords:** Allergic rhinoconjunctivitis, Nutraceutical, *Perilla frutescens*, Quercetin, Vitamin D3, Preventive treatment, Exacerbation

## Abstract

**Background:**

Lertal®, an oral nutraceutical, contains extract of Perilla, quercetin, and Vitamin D3. The current polycentric, randomized, parallel-group, controlled study aimed in the Phase II to evaluate the efficacy and safety of Lertal® in preventing allergic rhinitis (AR) exacerbations in children after the end of the pharmacological treatment phase.

**Materials and methods:**

One hundred twenty-eight children completed Phase II. Sixty-four children continued Lertal® treatment (Lertal® Group: LG) and 64 ones did not assume any medication (Observation Group: OG) for 4–12 weeks.

The study endpoints were the number, intensity, and duration of AR exacerbations, and the length of symptom-free time.

**Results:**

Children of LG halved the risk (HR = 0.54) of having AR exacerbation. Children of LG had significantly (*p* = 0.039) less AR exacerbations than OG children. In children with AR exacerbations, the total number of days in which each patient took at least one rescue medication was significantly (*p* = 0.018) lesser in LG children than OG ones. In the global population, the cumulative days treated with rescue medication was significantly (*p* < 0.0001) higher in OG than in LG. There was no clinically relevant adverse event.

**Conclusions:**

The present study documented that prolonged Lertal® assumption was safe and able to significantly reduce, such as halving, the risk of AR exacerbation, their duration and the use of rescue medications, after the suspension of the one-month antihistamine treatment. Therefore, Lertal® could be envisaged as an effective preventive treatment in AR children able to guarantee long symptom-free time.

**Trial registration:**

Clinical trial registration: ClinicalTrials gov ID NCT03365648.

## Background

Allergic rhinoconjunctivitis (AR) is the most common IgE-mediated disorder [1]. Children suffering from AR present nose (itching, sneezing, rhinorrhoea, and congestion) and eye (itching, redness, lacrimation, and eyelid swelling) symptoms. Interestingly, AR may also affect mood, sleep, leisure activity, and scholastic performance. AR symptoms depend on allergic inflammation that is characterized by a typical eosinophilic infiltrate [2].

Antiallergic drugs, antihistamines (oral or nasal) and nasal corticosteroids, represent the standard therapy for AR. However, these drugs may exert symptomatic relief of symptoms without persistent improvements of allergic disorder [3, 4]. In addition, their discontinuation is associated with quick symptom and cellular infiltrate relapse. It has been reported that symptoms and cellular infiltrate reappeared after suspension of an intranasal corticosteroid treatment: respectively after 3 days for symptoms, after 4 days for nasal neutrophils, and 6 days for eosinophils [5]. On the other hand, aggressive therapy and prolonged use of medications could induce significant side effects in children [6]. Therefore, there is increasing interest for new treatments for children with AR [7]. In this regard, there is evidence that nutraceuticals may be combined with standard therapy to speed up recovery, make it long lasting, avoid aggressive therapeutic regimens, and potentially prevent clinical relapse [8, 9].

Lertal® is a novel oral food supplement, containing *Perilla frutescens*, quercetin, and vitamin D3. The dry seed extract of *Perilla frutescens* contains rosmarinic acid and other flavonoids, such as luteolin, apigenin and chrysoeriol, and has shown in vivo and in vitro potential anti-allergic activity [10, 11]. Quercetin tends to stabilize cell membranes and block degranulation of mast cells and basophils, inhibiting the release of pro-inflammatory mediators and cytokines implicated in allergic inflammation [12, 13]. Vitamin D3 is essential for the normal function of the immune system and may exert a role in both prevention and potential treatment of AR, restoring physiological T regulatory activity and exerting also anti-inflammatory activity [14–16].

Notably, Lertal® is formulated in bilayer tablets composed of a fast-release layer that allows the rapid antihistamine activity of *Perilla*, and a sustained-release layer that enhances Quercetin and Vitamin D3 bioavailability and anti-allergy activity spread for long time.

Lertal® has been recently found to be able to reduce symptom severity and medication use in adults with AR [17]. As that study was open and conducted in adults, two hypotheses were therefore tested in a paediatric model of children with AR: i) the Lertal® capability to sustain the effectiveness of antihistaminic therapy and/or reduce its insensitivity, and ii) the Lertal® potentiality to prevent possible relapse after antihistamine therapy suspension. Therefore, a polycentric, randomized, study was designed in two phases. The first phase was conducted as double blind, placebo-controlled trial during standard antihistaminic AR treatment and evaluated the efficacy and safety of Lertal® as an add-on treatment. The obtained findings demonstrated that Lertal® was able to significantly prevent the occurrence of clinical worsening during the active treatment and was safe in AR poly-allergic children [18]. The second phase was designed as open and parallel-group study and was conducted after the end of the blind-period. Therefore, we currently report the outcomes of the second phase of this trial.

## Materials and methods

The phase II was an open-label, parallel-group, extension study in which patients treated with study product in Period I continued treatment with Lertal® tablets, whereas patients initially treated with placebo received no further treatment [18]. One hundred and sixty patients suffering from AR were planned for enrolment in 17 Italian Paediatric Allergy clinics. AR diagnosis was performed, according to validated criteria [3, 4], such as if nasal symptom history was consistent with documented sensitization.

Inclusion criteria were: age range 6–12 years, AR diagnosis, sensitization to house dust mites or pollens, Total Symptoms Score (TSS) ≥ 15 and at least 1 for nasal congestion, written informed consent of patients and of parents or legal guardians. Exclusion criteria were: uncontrolled asthma, secondary rhinitis to other causes, concomitant acute or chronic rhinosinusitis, nasal polyps, current use of topical or systemic corticosteroids, antihistamines, antileukotrienes, inadequate washout of them, nasal anatomic defect, respiratory infections in the last 2 weeks, participation in other clinical studies in the last month, documented hypersensitivity to the study product or its excipients, and trip planned outside of the study area.

TSS was the sum of the total nasal symptom score (TNSS), including itching, sneezing, rhinorrhea, nasal congestion, plus the total ocular symptom score (TOSS), including itching, hyperaemia of conjunctiva, tearing, plus total throat symptom score (TTSS), including itching and coughing. With the help of their parents, patients scored symptoms severity on a 4-point scale: 0 = absent or irrelevant, 1 = mild, 2 = moderate, 3 = severe.

After the 4-week active treatment period, children treated with Lertal® plus standard therapy continued to take Lertal® tablets (1 tab/day for 4–12 weeks) alone (such as without antihistamines), whereas children treated with Placebo suspended any treatment. The current treatment lasted 4 weeks in children with pollen allergy, whereas 12 weeks in children with perennial allergy.

Each Lertal® tablet contains the following active ingredients in a double-layer “fast-slow” release tablets: Quercetin 150 mg, *Perilla frutescens* 80 mg (as dry extract of the seeds containing rosmarinic acid, luteolin, apigenin and chrysoeriol), and Vitamin D3 5 mcg (200 IU).

Treatment with systemic or intranasal corticosteroids, leukotriene antagonists, and sodium cromoglicate were prohibited during the study. Two visits, were scheduled during this period to collect efficacy, safety and quality of life data. Patients should return their diaries at these visits in order to collect data concerning exacerbations and or adverse events. Patients also need to return all unused product to check compliance with treatment.

Patient-reported outcomes and diary recorded data, including the symptom relapse, the use of rescue medication/concomitant medication and the occurrence of adverse events, were collected at the visits. Clinical data were reported in an electronic Case Report Form (eCRF).

The end-points of the Phase II were the number, intensity, and duration of AR exacerbations, and the length of symptom-free time. Exacerbation was defined as the need of restarting an antihistamine medication of any kind, at any dose and of any duration because of symptom recurrence.

Patients were visited at the beginning and at the end of Phase II, such as after 4 weeks for children allergic to pollens and 8 weeks for children allergic to mites.

Safety was assessed on the incidence of adverse events for each treatment and on physical examinations.

The study protocol was approved by the Ethics Committees of each center. The study was registered at ClinicalTrials.gov ID NCT03365648.

The between-group analysis was performed by means of a t-test for independent samples or analogous non-parametric test or a Chi-square test in contingency tables. The Hazard Ratio (HR) was calculated by the univariate Cox model and Kaplan-Meier curves were performed. The time to first exacerbation was calculated as days elapsed between the start of period II (i.e. unblinding date) and the start date of the first exacerbation or the last date of observation. In particular, a reduction in the rate of first AR exacerbation of approximately 30%, corresponding to a HR of 0.70, was considered clinically relevant [19]. Missing data were substituted by worse case method.

## Results

Globally, 146 children (94 males and 52 females, aged 6–12 years, mean age 9.1 + 1.88) were randomized in the Phase I of the study. There were 18 children who dropped out. The Phase II study included a total of 128 patients, of which 64 assigned to open Lertal® therapy (Lertal® Group: LG) and 64 to observation alone (Observation Group: OG).

The two groups were homogeneous as far as age, gender, BMI, type of allergy, time from diagnosis and symptom severity are concerned at baseline.

**Fig. 1:**
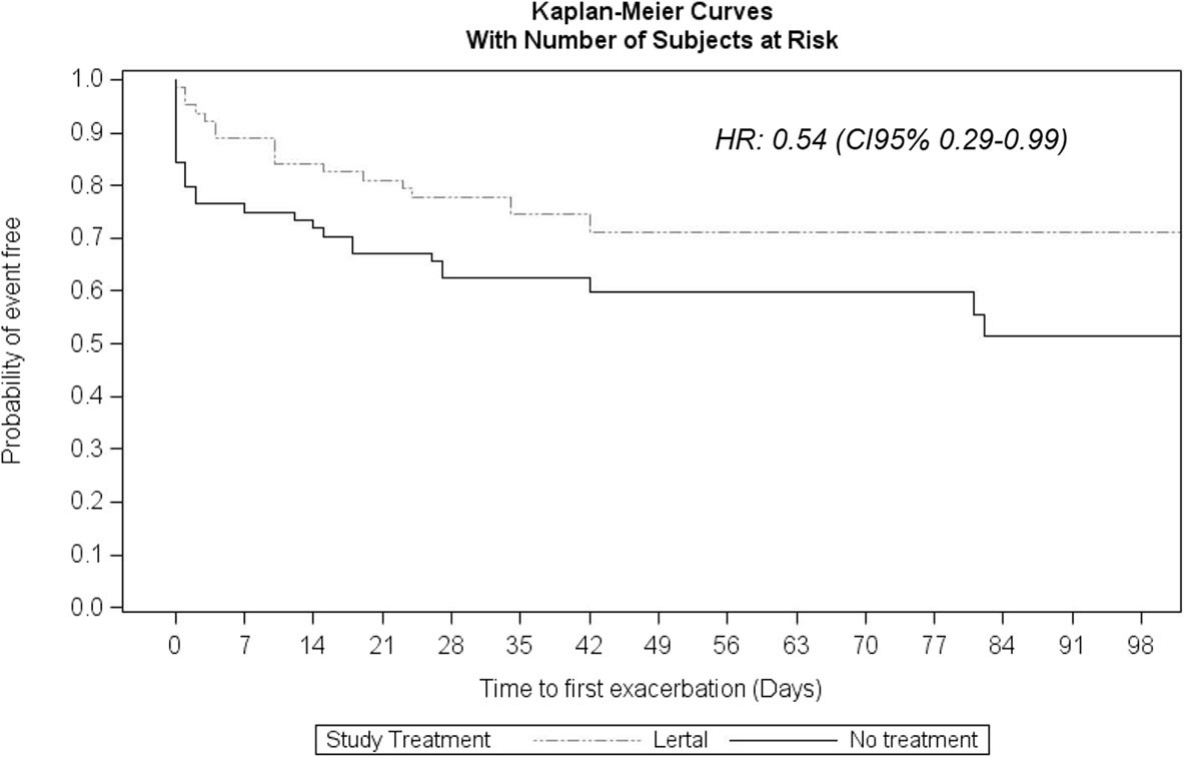
Kaplan-Meyer Plot. Time to first exacerbation calculated as days elapsed between the start of Phase II (i.e. unblinding date) and the start date of the first exacerbation or the last date of observation in Lertal® Group and Observation Group

The LG showed a significant difference concerning the duration of symptom-free days in comparison with OG (Log-Rank test = 4.16; *p* = 0.0413) with a HR 0.54 (CI 95% 0.29–0.99), as shown in Fig. 1.

**Fig. 2:**
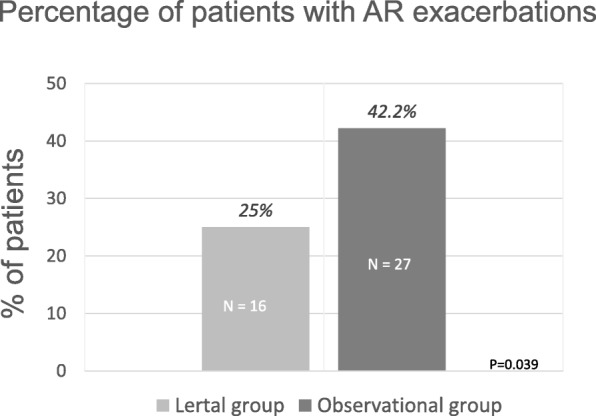
Percentages of children experiencing AR exacerbation in Lertal® Group and Observation Group

Considering the number of children who experience an AR exacerbation, there was a significant difference between groups as only 16 children (25%) in the LG had an AR exacerbation, whereas 27 children (42.2%) of OG had an AR exacerbation (*p* = 0.039), as shown in Fig. 2. Analysing only the children with AR exacerbation, the total number of days in which each patient took at least one rescue medication was significantly (*p* = 0.018) lesser in LG than OG (9.6 + 9 days and 28.5 + 27.2 days respectively). Considering the global population, the cumulative days treated with rescue medication was significantly (*p* < 0.0001) higher in OG than in LG (683 days and 153 days respectively).

Analysing only children treated with concomitant medications, LG children had tendentially less AR exacerbations than OG children (7 vs 12; *p* = 0.051).

Lertal® treatment was well tolerated and no serious adverse event was reported. Out of the patients who entered in Period II, 55 patients experienced one or more adverse events (32 in LG and 23 in CG). One patient in LG discontinued because of acute urticaria and cough due to pollen allergy exacerbation.

## Discussion

AR affects up to 40% of the general population [1], and represents a relevant burden for the society. Adequate, effective, and safe treatment is always recommended in all AR patients, but with particular attention in children. A particular aspect of allergic disorders is that medications do not cure AR, but limit to relief symptoms and control inflammation. Notably, the results obtained by long-term continuous anti-inflammatory treatments, when suspended, rapidly disappear and are not able to modify the natural history as demonstrated by an elegant study in pre-schoolers with wheezing [20]. In fact, the PEAK (prevention of early asthma in kids) study evidenced that 2-year inhaled fluticasone did not modify the further progression of the disease.

AR is characterized by type-2 immune response that promotes, sustains, and maintains an abundant eosinophilic infiltrate in the nasal mucosal tissue and the ongoing production of allergen-specific IgE. The link between the inhaled causal allergen and IgE expressed on the surface of mast cells starts a cascade of inflammatory events, mainly concerning the histamine (the pivotal mediator in AR) release that result in symptom occurrence. For this reason, antihistamines and intranasal corticosteroids are the most effective and used medications in AR patients [1, 3, 4]. However, the clinical effect of a single dose of an antihistamine usually lasts until 24–36 h, then symptoms reappear promptly [21]. Similarly, the duration of intranasal corticosteroids effects is very short-lived after suspension, such as in a few days symptoms and inflammatory events recur [5]. In addition, both antihistamines and intranasal corticosteroids may be unable to completely inhibit allergic reaction in some circumstances, such as highly allergic patients, intense allergen exposure, interfering disorders. Therefore, the use of add-on medications could be fruitful in such situations. Actually, the Phase I of the current study demonstrated the Lertal® used as add-on treatment was able to significantly reduce the symptom worsening, i.e. AR exacerbation, in poly-allergic children [18]. Interestingly, this favourable effect was evident in the second part of the active treatment, such as between the third and the fourth week, when some patients, after an initial response to drug treatment, showed a symptom worsening. This preventive activity could be explained by the anti-inflammatory, the immune-modulatory, and the anti-allergic properties of the *Perilla* extract, the quercetin, and the vitamin D3.

The outcomes of the Phase II not only confirm indeed the favourable effects observed in the Phase I, but also highlight an even more relevant preventive activity consequent to the prolonged use. In particular, we would underline two main issues. The highly favourable HR value of 0.54: it means that the risk of AR exacerbation had reduced in children taking Lertal® for a 4–12-week period of 46% in comparison with children without preventive intervention after the suspension of the standard 4-week antihistamine treatment. This finding is consistent with previous studies conducted in patients with asthma and in children with allergic rhinitis. Tonelli and colleagues demonstrated that 56% of asthmatic patients, regularly treated with inhaled corticosteroids for at least 6 months, experienced asthma recurrence in the first month after discontinuation of the pharmacological therapy [22]. A randomized and placebo-controlled study evaluated the efficacy of allergen immunotherapy in preventing asthma exacerbations during an inhaled corticosteroid reduction period [23]. Compared with placebo, there was a reduced risk of an exacerbation with deterioration in asthma symptoms (HR 0.64) in the group taking the higher dose of allergen. Another randomized and placebo-controlled study investigated the effect of allergen immunotherapy on the risk of developing asthma in a large group of children with AR [24]. The sublingual immunotherapy reduced the risk of experiencing asthma symptoms or using asthma medication at the end of the trial (OR = 0.66) and during the 2-year follow-up. Notably, the primary endpoint, such as the time to onset asthma, was not different between groups.

Therefore, the present study provided a clinically relevant results, also consisting with these studies, such as Lertal® treatment was able to approximately halve the risk of AR exacerbation after one-month antihistamine treatment. This outcome is also supported by the larger number of Lertal®-treated children (75%) who did not experience AR exacerbation than untreated children (58%). In addition, these findings are consistent with outcomes documented in the Phase I that evidenced that Lertal®, used as add-on therapy, was able to tendentially improve the effect of the standard AR treatment and especially Lertal® significantly reduced the possible occurrence of intercurrent relapse during the standard treatment in children with AR. In addition, it has been evidenced that Lertal® was also able to significantly reduce the number of exacerbations, the duration, and the use of rescue medications. Notably, the number of symptom-free days was significantly higher in Lertal®-treated children. This outcome has a clinical relevance of paramount importance as it underlines the possibility of avoiding the use of medications, mainly after a long antihistamine course, i.e.4 weeks. This point deserves adequate attention as AR cannot be cured by medications, even though prescribed for long periods. Therefore, reducing the use of pharmacological therapy is particularly required in childhood. In addition, this outcome deserves also a consideration from a pharmaco-economic point of view. Actually, a greater number of symptom-free days means a significantly saving of medication use. Last but not least, symptom-free condition is closely associated with a good quality of life, as it is well known that rhinitis severity affects this aspect.

These findings may be explained by a sustained and prolonged effect on allergic reaction depending on the multifaceted mechanisms of action exerted by Lertal®. In particular, Lertal® effects seem to be grounded in the complex anti-inflammatory, immune-modulatory, and anti-allergic activity exerted on the immune response by the three compounds.

However, this study has some limitations, including the lack of measurement of inflammation biomarkers and of objective functional assessment. In addition, it has to be noted that the Vitamin D3 supplementation may be controversial as recently pointed out [25]. Wjst contested the indiscriminate use of this pre-hormone in childhood as there are no convincing evidence about its real effectiveness. However, there are substantial data supporting its utility in many disorders, including childhood asthma. In this regard, Litonjua has recently stated that evidence continues to accumulate that vitamin D supplementation helps to prevent the development of asthma and recurrent wheeze in early life, and may also help in the management of asthma [26].

On the other hand, the strength of this study was the methodological accuracy, based on the double-blinded, randomized, parallel-group, and placebo-controlled design of the Phase I, the presence of a successive observational period (Phase II), and the sample size estimate.

## Conclusions

The present study documented that prolonged Lertal® treatment was safe and able to significantly reduce, such as halving, the risk of AR exacerbation occurrence, their duration, and the need of symptomatic medications in children with allergic rhinitis. Therefore, Lertal® could be envisaged as an effective preventive treatment in AR children able to guarantee long symptom-free time.

## Data Availability

All data analysed during this study are included in this published article and its supplementary information files.

## Abbreviations

AR: Allergic Rhinitis
CRF: Case report form
HR: Hazard ratio
IgE: Immunoglobulin E
LG: Lertal® Group
OG: Observation Group
TSS: Total symptom score
